# Explainable Remaining Tool Life Prediction for Individualized Production Using Automated Machine Learning

**DOI:** 10.3390/s23208523

**Published:** 2023-10-17

**Authors:** Lukas Krupp, Christian Wiede, Joachim Friedhoff, Anton Grabmaier

**Affiliations:** 1 Fraunhofer Institute for Microelectronic Circuits and Systems, 47057 Duisburg, Germany; 2 CAX Technologies, University of Applied Sciences Ruhr West, 45407 Mülheim an der Ruhr, Germany; 3 Department of Electronic Components and Circuits, University of Duisburg-Essen, 47057 Duisburg, Germany

**Keywords:** automated machine learning, explainable artificial intelligence, free-form machining, individualized production, remaining useful lifetime, tool condition monitoring

## Abstract

The increasing demand for customized products is a core driver of novel automation concepts in Industry 4.0. For the case of machining complex free-form workpieces, e.g., in die making and mold making, individualized manufacturing is already the industrial practice. The varying process conditions and demanding machining processes lead to a high relevance of machining domain experts and a low degree of manufacturing flow automation. In order to increase the degree of automation, online process monitoring and the prediction of the quality-related remaining cutting tool life is indispensable. However, the varying process conditions complicate this as the correlation between the sensor signals and tool condition is not directly apparent. Furthermore, machine learning (ML) knowledge is limited on the shop floor, preventing a manual adaption of the models to changing conditions. Therefore, this paper introduces a new method for remaining tool life prediction in individualized production using automated machine learning (AutoML). The method enables the incorporation of machining expert knowledge via the model inputs and outputs. It automatically creates end-to-end ML pipelines based on optimized ensembles of regression and forecasting models. An explainability algorithm visualizes the relevance of the model inputs for the decision making. The method is analyzed and compared to a manual state-of-the-art approach for series production in a comprehensive evaluation using a new milling dataset. The dataset represents gradual tool wear under changing workpieces and process parameters. Our AutoML method outperforms the state-of-the-art approach and the evaluation indicates that a transfer of methods designed for series production to variable process conditions is not easily possible. Overall, the new method optimizes individualized production economically and in terms of resources. Machining experts with limited ML knowledge can leverage their domain knowledge to develop, validate and adapt tool life models.

## 1. Introduction

With the advancement of Industry 4.0, the demand for highly customized products is increasing. A growing proportion of single-part and small-batch production manifests this in the manufacturing industry. The resulting frequent machine and process reconfigurations increase the susceptibility to process errors, which is unacceptable for applications requiring a high product quality and reliability. Die making and mold making combine high product quality requirements and a dominant share of individualized production [1,2]. The core technology in die making and mold making is machining, particularly multi-axis milling, for manufacturing complex free-form workpieces [3]. The decisive quality parameters are their dimensional accuracy and surface roughness, which are significantly influenced by the wear of the cutting tools [4,5,6]. Even minor deviations from the specification can lead to defective end products, e.g., in injection molding or die-casting processes. Therefore, monitoring the machining processes and tool wear is essential to avoid scrap and rework [7].

In recent years, remaining tool life prediction based on sensor-driven process monitoring has been increasingly investigated in this context [8]. The remaining tool life prediction enables a joint estimation of the current tool condition based on the monitoring data and the duration until quality-related specifications of the process are violated. Thus, the quality and productivity of machining processes become controllable and the job-shop scheduling is simplified due to increased plannability [9]. Simultaneously, process-integrated sensors allow for reductions in time-consuming measurements using manufacturing metrology.

However, single-part and small-batch production conditions have made the development of remaining tool life prediction methodologies considerably difficult [10]. In particular, frequent changes in the workpiece geometries and process parameters do not allow for the direct inference of the tool condition from the sensor data due to a lack of comparability. Furthermore, the prediction of the remaining tool life is affected by the increased uncertainty regarding future process conditions. Therefore, previous approaches mainly focus on series production under constant process conditions, implying that the used prediction models are not adaptable.

This paper aims to develop and investigate a methodology enabling the remaining tool life prediction for individualized production, i.e., single-part and small-batch production. The prediction should be based on process-integrated sensors to ensure permanent process monitoring under constantly changing workpieces and cutting parameters.

The contributions of our work include the following:

1. A remaining tool life prediction methodology adaptable to new process conditions without manual intervention through automated machine learning (AutoML) while jointly explaining the predictions for model validation and optimization;
2. A new dataset and the methodology for its generation, representing gradual tool wear and its influence on the workpiece surface in individualized production under continuous variation in workpieces and cutting parameters;
3. A detailed evaluation of the methodology based on the new dataset, comparing it with a state-of-the-art approach for series production and investigating its explanation and generalization capabilities as well as its potential to increase the prediction robustness.

This paper is structured as follows. Section 2 provides an overview of the background and current state-of-the-art methods in the field of remaining tool life prediction, focusing on approaches based on sensor-driven process monitoring. Section 3 introduces the new remaining tool life prediction methodology for individualized production and highlights its three major components. Section 4 outlines the implementation details of the methodology and describes the dataset generation to evaluate it. In Section 5, the evaluation procedure is presented, and the results are discussed. Section 6 summarizes and concludes the paper.

## 2. Related Work

The state-of-the-art methods in sensor-driven remaining tool life prediction comprise two main approaches: direct [11] and criterion-based [12]. Direct methods use models generating a temporal output from the process-describing sensor data. The tool state and the decision threshold regarding the end of the tool life are, therefore, only implicitly part of the model and cannot be extracted or set separately. Criterion-based methods integrate an intermediate step via a tool life criterion to indicate the tool condition. The subsequent extrapolation of the tool life criterion allows for the setting of arbitrary decision thresholds. In addition, the tool condition is directly available for further applications, e.g., for integration into a simulation. Due to its comprehensive significance for the process quality, the tool condition and the end of tool life are usually determined based on tool wear [13]. Alternatively, quality parameters, such as the workpiece surface roughness, can be used as tool life criteria.

The data basis for the remaining tool life models is generated using the state-of-the-art sensor types in process monitoring [8]. The monitoring variables are the cutting force [14], vibration [15], drive current and power [16] or machine tool controller signals [17]. Since the sensor data provide the input for the predictions, either purely data-based [18] or hybrid physics- and data-based models [19] are used. In the area of data-based remaining tool life prediction, machine learning (ML) models and particularly neural network architectures, like convolutional neural networks (CNNs) [20], temporal convolutional networks (TCNs) [21] or long short-term memory (LSTM) networks [22], are current research topics due to their high adaptivity, accuracy and suitability for temporal predictions.

**Figure 1:**
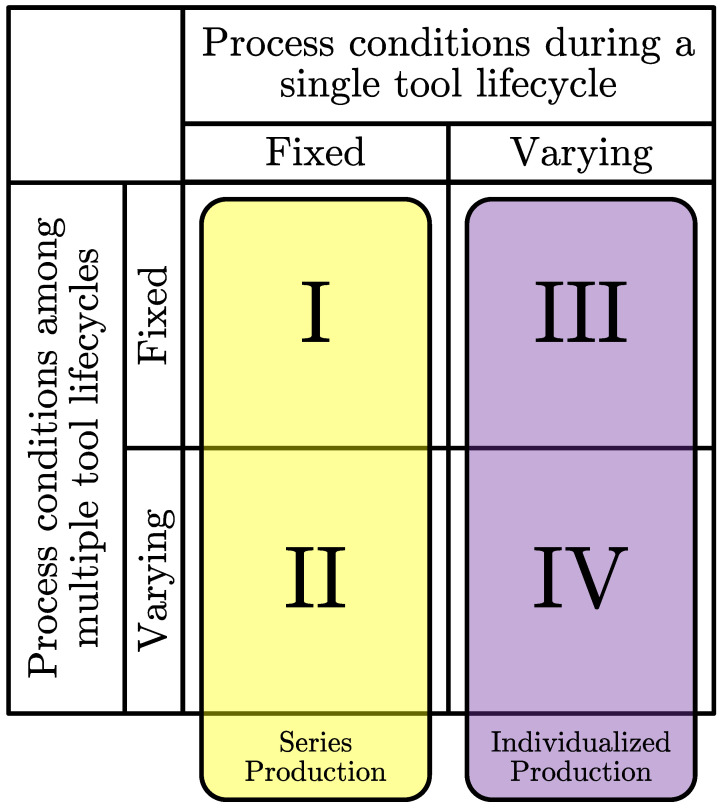
Possible dataset variants in the area of remaining tool life prediction depending on the degree of process condition variations during single and multiple tool life cycles.

Their underlying production scenarios and datasets are the most significant distinguishing characteristics of the prediction models. A remaining tool life dataset comprises the sensor and target data over the life cycle of multiple tools. Possible variants of datasets are shown in Figure 1, depending on the respective degree of process condition variations during single and multiple tool life cycles. Process conditions refer to influencing factors, i.e., the tool shape and material; workpiece shape and material; cutting parameters; machine tool design and its condition or tool path; and process kinematics. While the dataset variants I, e.g., Ref. [23], and II, e.g., Ref. [24], mainly represent series production, the combinations III [25] and IV describe the individualized production scenario.

Previous work on sensor-driven remaining tool life prediction mainly investigates series production scenarios [8]. Individualized production, i.e., the variation in process conditions during a tool life cycle, is hardly considered. A single approach analyzes varying cutting parameters during the tool life cycle [25]. However, the same workpiece is manufactured repeatedly. In [9], a methodology for small-batch production is developed using a dataset containing several identical cutting operation sequences. The approach of Matsumura et al. [26], while considering varying workpieces during the tool life cycle, requires direct wear measurements and is thus outside the scope of sensor-driven predictions.

Overall, sensor-driven remaining tool life prediction has not yet been analyzed under variable cutting parameters and workpiece geometries during single and multiple tool life cycles. Datasets according to variant III, like [25], do not include the implied degree of process condition variation, and datasets according to variant IV do not exist. Therefore, it is still unknown whether the feature extraction methodologies for sensor data under fixed process conditions are applicable. Furthermore, previous remaining tool life prediction models do not mitigate the uncertainty due to variable future process conditions.

Several papers investigate the use of AutoML methods to make the benefits of ML-based models even easier to apply to tool condition monitoring [6,27,28,29]. AutoML leverages the autonomous adaptation of models to changing process conditions, especially in individualized production. However, with increased autonomy in the generation of models, their validity must be ensured. Although the first approaches to explaining ML models in the context of machining process monitoring exist [30,31,32,33], methods combining AutoML and model explainability are missing so far.

## 3. Methodology

Our methodology extends the state-of-the-art methods described in the following three points to enable and overcome the challenges of the remaining tool life prediction for individualized production: an extended and non-invasively acquired feature set as input to the prediction model (Section 3.1); a new AutoML-based prediction model incorporating future feature estimates (Section 3.2); and a feature importance ranking algorithm that exploits the AutoML-based nature of our prediction model to explain its output (Section 3.3).

### 3.1. Sensors Signals and Feature Extraction

The model inputs are features obtained from the data of process-integrated sensors. The characteristics of individualized production must be considered when selecting the sensors. There are frequent changes in process-related components, such as the tool, the tool holder or the clamping device. In addition, different machine types are used due to the variety of workpieces. Accordingly, permanent and comprehensive process monitoring is only possible if sensors that are neither directly dependent on the process-related components nor the machine tool are used.

**Figure 2:**
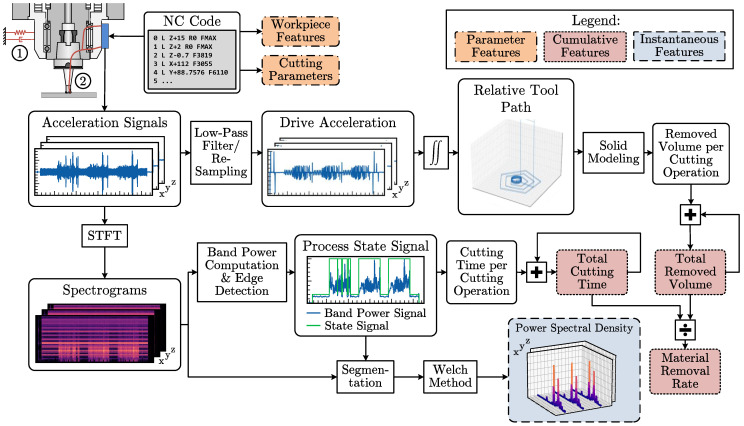
Sensor system architecture showing the signal processing and feature extraction steps based on three spindle-mounted accelerometers covering the spatial axis. The accelerometers acquire the mass-dependent vibration of the spindle due to the cutting forces ➀ and the process-related structure-borne sound ➁.

Vibration sensors fulfill this requirement and allow a trade-off between process proximity and independence. Due to the property of vibration propagation via the machine structure, sensors can be placed on machine components that are only indirectly involved in the process, such as the spindle. If the sensor sensitivity is sufficient, it is still possible to detect process emissions with a high resolution. Figure 2 shows the sensor system and signal processing approach followed in this paper based on these considerations.

To ensure a high degree of process information, both the mass-dependent vibration of the machine component due to the cutting forces and the process-related structure-borne sound, marked as ➀ and ➁ in Figure 2, are acquired. State-of-the-art micro-electro-mechanical system (MEMS) accelerometers for industrial condition monitoring enable this, featuring low noise, a high sensitivity and a high bandwidth. In addition, their small size and high energy efficiency make them suitable for use in embedded systems on dynamic machine components. The signals from the acceleration sensors on the three spatial axes form the basis for feature extraction.

In total, three types of features are distinguished: instantaneous, parameter and cumulative features. Instantaneous features are time- or frequency-domain features describing the current milling process at the signal level and are therefore derived directly from the sensor data. They contain information about the tool state at the current time but are also influenced by the process conditions, i.e., in particular, cutting parameters and workpiece geometry or the resulting engagement conditions. In previous work, for the most part, only instantaneous features were used due to the assumed constant process conditions per tool life cycle. Since process conditions are constantly changing in individualized production, it becomes difficult to distinguish the tool condition based on instantaneous features only. Therefore, in this paper, we investigate parameter features as model inputs in addition to the instantaneous features. The parameter features describe the process parameters set during process planning, i.e., in particular, cutting parameters, such as the feed per tooth $f_{z}$, the cutting speed $v_{c}$ and the axial depth of cut $a_{p}$ or the geometry parameters of the workpieces. We assume that the parameter features can be extracted from the numerical control (NC) code, which represents a second machine- and process-independent data source in addition to the acceleration sensors. The NC code is transferred to the sensor system from the computer-aided manufacturing (CAM) software running on a dedicated computer or in the cloud.

The cumulative features are process-describing variables summed over time, such as the tool’s cutting time or the volume of material already removed. Due to their resulting monotonic behavior, the cumulative characteristics correlate very well with potential target variables that are also monotonic, such as gradual tool wear.

The basis for feature extraction is the short-time Fourier transform (STFT), generating time-frequency spectrograms from the acceleration signals. The spectrograms are converted into a process state signal by determining the band power in the dominant frequency range of the machining process. Then, an edge detection algorithm is applied to the band power signal to receive the state signal. The process state signal enables the logical segmentation of continuous cutting operations. For each cutting operation, using the Welch method, the instantaneous features in the form of the power density spectra of the triaxial signals are determined from the spectrograms. It is known that the power density spectra as instantaneous features in series production scenarios show good correlations with tool wear [30,34]. Furthermore, the cutting time per cutting operation is calculated based on the process state signal. The cumulative feature of the total cutting time of the tool is then derived by accumulating the cutting time per cutting operation.

The low-frequency drive acceleration signals are extracted from the broadband acceleration signals via low-pass filtering and resampling in a second signal processing path. The drive acceleration signals can be converted into relative position information of the axes via double integration with intermediate error correction. If the tool geometry is known, solid modeling allows for an estimation of the removed material volume per cutting operation via Boolean operations [35]. The cumulative feature of the total removed volume is derived by accumulating the removed volume per cutting operation. The quotient of the total removed volume and the total cutting time of the tool represents the material removal rate. The material removal rate is a measure of the process productivity. Since the feature is derived from two cumulative features, it is also attributed to the cumulative features.

### 3.2. Prediction Model

**Figure 3:**
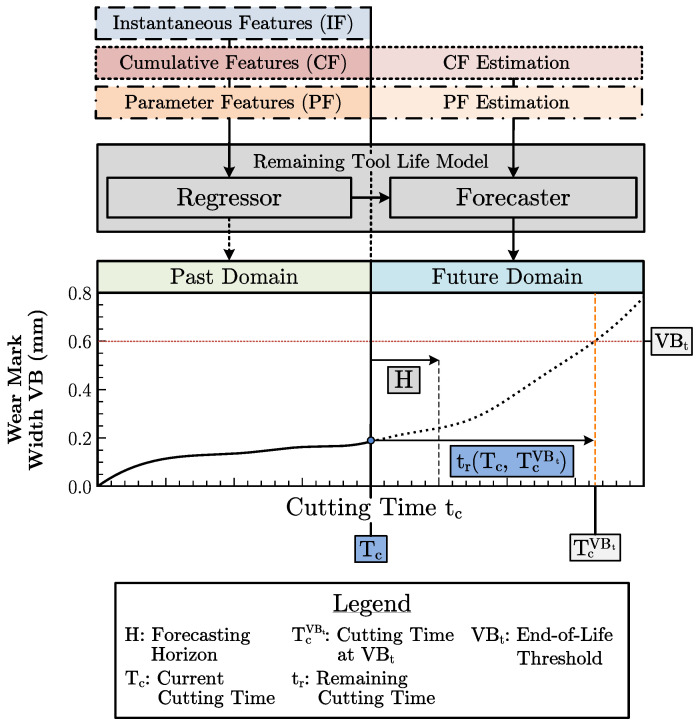
Remaining tool life prediction methodology based on an extended feature set. The feature set contains not only instantaneous features with information on the current tool state but also cumulative and parameter features with context information on current and past processes. Additionally, the model allows for estimates of future features as inputs to include a priori knowledge.

In this paper, the criterion-based approach shown in Figure 3 is followed for the remaining tool life prediction. Since the workpiece surface roughness and dimensional accuracy are mainly influenced by the flank wear of the milling tool, the wear mark width VB is considered the primary tool life criterion in the following. In this paper, the VB is defined as the average of the maximum wear mark widths per cutting edge. The procedure to determine the VB in practice is described in Appendix A. The criterion-based approach allows for an arbitrary choice of the end-of-life threshold, i.e., the tool wear threshold ${\mathrm{VB}}_{t}$. The end-of-life threshold is critical in individualized production since it may change depending on the required workpiece tolerances.

The goal is to predict at the current cutting time step $T_{c}$ the future time step $T_{c}^{{\mathrm{VB}}_{t}}$ where the wear threshold value ${\mathrm{VB}}_{t}$ is reached. The remaining tool life $t_{r}$ can then be derived according to Equation  (1):

(1) $$t_{r}\left(T_{c},T_{c}^{{\mathrm{VB}}_{t}}\right)=T_{c}^{{\mathrm{VB}}_{t}}-T_{c}$$

The criterion-based prediction approach represents a multi-series forecasting problem, with the particularity that a regression of the tool state from the sensor data features must first be performed to perform the forecast. Accordingly, the model shown in Figure 3 includes a regression and a forecasting component. The dataset D for training and testing the model contains *N* tool instance series and is thus defined as $D={\left\{D_{i}\right\}}_{i=1}^{N}$. The samples of $D_{i}$ are defined as $D_{i}=\left\{X_{i,1:T_{c}}^{\left(p\right)},X_{i,T_{c}+1:T_{c}+H}^{\left(f\right)},y_{i,1:T_{c}},y_{i,T_{c}+1:T_{c}+H}\right\}$ with $T_{c}$ being the instantaneous cutting time step beyond which the wear curve $f_{\mathrm{VB}}$ is to be forecasted and *H* being the forecasting horizon, i.e., the cutting time steps over which a model predicts the $f_{\mathrm{VB}}$. The tensors $X_{i,1:T_{c}}^{\left(p\right)}$ and $X_{i,T_{c}+1:T_{c}+H}^{\left(f\right)}$ describe the past features and estimated future features. The vectors $y_{i,1:T_{c}}$ and $y_{i,T_{c}+1:T_{c}+H}$ describe the past and future targets. For simplicity, the index of the tool instance *i* is omitted in the following.

An abstracted time step in the model refers to a continuous cutting operation *j*. Hence, each time step can be assigned a sample of the form $\left\{x_{j},y_{j}\right\}$, with $x_{j}$ being the feature vector and the scalar $y_{j}$ being a wear value. In contrast to the state-of-the-art methods, the prediction approach shown in Figure 3 is based on inputs from past cutting operations and allows for the integration of estimates of future features. Thus, the constantly changing conditions of individualized production can be considered. According to Equations (2a) and (2b), the feature tensors of past cutting operations $X_{1:T_{c}}^{\left(p\right)}$ are composed of the instantaneous features $f_{i}$, the parameter features $f_{p}$ and the cumulative features $f_{c}$. The features are obtained based on the methodology introduced in Section 3.1.

(2a) $$\begin{array}{c} X_{1:T_{c}}^{\left(p\right)}=\left[x_{1},\ldots ,x_{T_{c}}\right] \end{array}$$

(2b) $$\begin{array}{c} x_{j}={\left[f_{i,j}^{T},f_{p,j}^{T},f_{c,j}^{T}\right]}^{T} \end{array}$$

The future feature tensors $X_{T_{c}+1:T_{c}+H}^{\left(f\right)}$ are based on estimates and prior knowledge about future manufactured workpieces and cutting parameters. Since the instantaneous features can only be generated based on a machining process that has taken place, only the parameter features ${\hat{f}}_{p}$ and cumulative features ${\hat{f}}_{c}$ according to Equations (3a) and (3b) can be considered for the future feature tensors.

(3a) $$\begin{array}{c} X_{T_{c}+1:T_{c}+H}^{\left(f\right)}=\left[{\hat{x}}_{T_{c}+1},\ldots ,{\hat{x}}_{T_{c}+H}\right] \end{array}$$

(3b) $$\begin{array}{c} {\hat{x}}_{j}={\left[{\hat{f}}_{p,j}^{T},{\hat{f}}_{c,j}^{T}\right]}^{T} \end{array}$$

Based on the feature tensors, the prediction is performed according to Figure 3. The presented model consisting of the regression component $f_{R}$ and the forecasting component $f_{F}$ can be formally described according to Equations (4a) and (4b).

(4a) $$\begin{array}{c} {\hat{y}}_{1:T_{c}}=f_{R}\left(X_{1:T_{c}}^{\left(p\right)},\theta^{R}\right) \end{array}$$

(4b) $$\begin{array}{c} {\hat{y}}_{T_{c}+1:T_{c}+H}=f_{F}\left(f_{R},X_{T_{c}+1:T_{c}+H}^{\left(f\right)},\theta^{F}\right) \end{array}$$

The tensors $\theta^{R}$ and $\theta^{F}$ represent the parameters of the prediction model, which are adjusted during training.

### 3.3. Explainablity Methodology

Given the prediction model and the dataset, the goal is to identify the features relevant to the model output to ensure model explainability. At the same time, the best possible model performance should be achieved, and the manual configuration effort during training, e.g., due to hyperparameter searches, should be minimized. Therefore, we pursue model explainability based on AutoML-driven training in this paper.

It is assumed in the following that the prediction model is generally represented by an ensemble of ML pipelines $M_{E}$. An individual ML pipeline $M_{\lambda }$ with the model parameters θ and hyperparameters λ describes the transfer from the input features to the final prediction. The pipelines are trained based on a training dataset $D_{train}$ generated from the previously introduced dataset D. Besides the adaptation of the model parameters θ, the training includes the combined architecture search and hyperparameter (CASH) optimization of the pipelines and a subsequent selection of several pipelines resulting in the ensemble $M_{E}$ [36]. The ensemble combines the predictions of the included pipelines to increase the prediction accuracy. It is assumed that all pipelines contain an importance score that they use for their internal feature selection to obtain an overall measure of feature importance. Algorithm 1 formally describes the procedure for generating a global feature importance measure to explain the model decision.
**Algorithm 1** AutoML-based combined remaining tool life prediction model generation and feature importance ranking**Input:** Pipelines $M_{\lambda }$ hyperparemeterized by λ∈Λ including feature selection based on importance score vectors s, Empirical generalization error function ${\hat{E}}_{G}$, Training dataset $D_{train}$, Training time budget *T*, Number *N* of pipelines to include in a final ensemble **Output:** Best-performing ensemble $M_{E}$ of pipelines, Global feature importance vector $s_{G}$
1:Solve $M_{\lambda^{*}}\in \underset{\lambda \in \Lambda }{\mathrm{argmin}}{\hat{E}}_{G}\left(M_{\lambda },D_{train}\right)$ s.t. $\sum t_{\lambda_{i}}<T$                 
▹ Standard time-bounded CASH optimization2:$M_{E}$ = **EnsembleSelection**$\left(M_{\lambda }^{*},D_{train},N\right)$                
▹ Standard weighted average ensemble method3:S = **CollectFeatureImportance**$\left(M_{E}\right)$    
▹ Returns a tensor containing the importance vectors of all pipelines in the ensemble4:$s_{G}=\frac{1}{N}\sum_{j=1}^{N}s_{j}$ with $S=\left[s_{1},\ldots ,s_{N}\right]$


Algorithm 1 is a global methodology, meaning that the feature importance output $s_{G}$ relates to the complete training dataset. However, the procedure can be extended without a loss of generality using state-of-the-art local algorithms, as evaluated in [37], to obtain importance scores for individual feature vectors.

## 4. Implementation

### 4.1. Sensor System

The sensor system implementation for analyzing the previously introduced remaining tool life prediction methodology requires the use of sensitive MEMS accelerometers as a core component, according to Section 3.1. Therefore, the CN-0549 platform from Analog Devices is used [38]. The platform consists of the ADXL1002 acceleration sensor with a 3 dB bandwidth of 11 kHz [39], the CN-0540 signal acquisition board [40] and the Cora-Z7 field-programmable gate array (FPGA) system-on-chip (SoC). The CN-0540 signal acquisition board features a 24-bit Σ-Δ analog-to-digital converter allowing sampling rates up to 256 kHz. A high sampling rate is required to support all milling applications, including high-speed scenarios with common spindle rotation speeds up to 60,000 rpm. With the maximum sampling frequency, a single spindle rotation is then still devoted to 256 sensor signal samples.

Furthermore, according to Section 3.1, the methodology requires the sensors to be mounted to the machine tool’s spindle. We assume that the spindle moves only along the three spatial axes. The principle is, therefore, only partially applicable in machines whose spindles can be tilted in addition to the translational movements. However, extending the sensor system with additional inclination sensors can restore the unrestricted applicability. A Hermle C30 U five-axis indexed milling center is used for further investigations. The machine tool’s spindle performs only translational movements and the machine table can be tilted and rotated. Thus, the machine tool fulfills the previous assumption. The ADXL1002 is a single-channel acceleration sensor. Therefore, three sensors are installed on the spindle to cover the spatial axes according to the setup shown in Figure 2.

### 4.2. Experimental Setup

**Figure 4:**
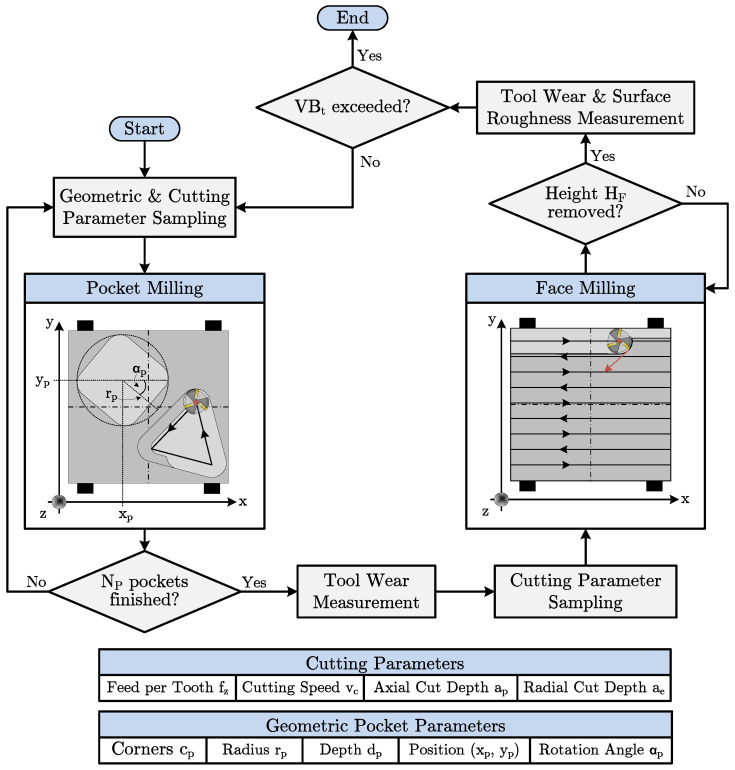
Methodology for the dataset generation based on a parameterizable pocket milling process. The dataset represents tool wear until exceeding an end-of-life threshold ${\mathrm{VB}}_{t}$ in individualized production scenarios under continuous variation in workpieces and cutting parameters.

To date, no dataset exists that represents tool wear in the context of individualized production with continuous variation in workpieces and cutting parameters. Therefore, in this paper, the methodology shown in Figure 4 is proposed for the dataset generation based on the pocket milling process.

The pocket milling process is selected due to the easily parameterizable workpiece geometries. The workpiece geometry is a polygon with a variable number of corners $c_{p}$. The variation in the number of corners allows for controllable variation in the engagement conditions of the cutter as its engagement angle changes along the tool path. The number of corners defines the maximum engagement angle. Furthermore, the radius of the polygon $r_{p}$, its depth $d_{p}$, its position as 2D coordinates $\left(x_{p},y_{p}\right)$ and its rotation angle $\alpha_{p}$ are varied. A computer-aided design (CAD) workpiece generator based on the geometry parameters is developed to sample the workpieces randomly. In addition to the workpiece geometries, the cutting parameters, i.e., the feed per tooth $f_{z}$, the cutting speed $v_{c}$ and the axial depth of cut $a_{p}$, are actively varied. The sampling of the cutting parameters is based on latin hypercube sampling (LHS) to cover the parameter space with the limited number of milling operations that can be performed during a tool’s life. The axial depth of cut $a_{e}$ varies automatically along the tool path due to the variable engagement angle.

A fixed number $N_{p}$ of pocket geometries are manufactured in a sequence. Subsequently, face milling removes the remaining material at a height $H_{f}$. The $H_{f}$ must be greater than the maximum pocket depth. The face milling process applies a zig-zag strategy and LHS-derived cutting parameters. After completing a sequence of pocket and face milling processes, the maximum wear mark widths of the cutting edges are measured and the VB is derived. In addition, the surface roughness of the workpiece is measured for reference after each face milling process. The combined pocket and face milling are repeated until the wear mark width exceeds a threshold ${\mathrm{VB}}_{t}$.

Since individualized production is dominantly represented in die making and mold making, the experimental requirements are based on industrial practice in this field. To manufacture the workpieces on an X155CrVMo12-1 steel cube with an edge length of 200 mm, a toroidal milling tool with three circular inserts (∅= 8 mm) is used. The processing is performed on the Hermle C30 U five-axis indexed milling center described in the previous section. The tool wear is measured using a Garant MM1 video measuring microscope and the workpiece surface roughness is measured using a MarSurf PS 10 device. Based on the sensor data obtained during the process execution and the NC code, the input features of the dataset are generated for each tool according to Section 3.1. Table 1 shows the components of the final input feature set. The maximum wear mark width VB averaged over the three inserts is used as the target. The detailed methodology to derive the VB is described in Appendix A. An end-of-life threshold of ${\mathrm{VB}}_{t}$ = 0.8 mm is selected based on the tool manufacturer’s recommendation. The milling process specifications, including the detailed cutting and workpiece parameter intervals, are summarized in Appendix B.

### 4.3. Model Implementation

**Figure 5:**
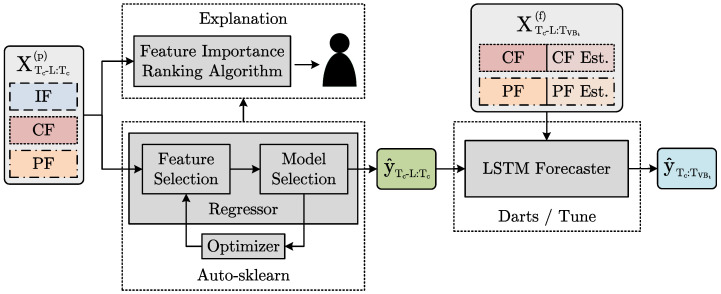
Framework implementing the automated and explainable remaining tool life prediction. The feature tensors $X_{T_{c}-L:T_{c}}^{\left(p\right)}$ and $X_{T_{c}-L:T_{c}^{{\mathrm{VB}}_{t}}}^{\left(f\right)}$, including the instantaneous features (IFs), cumulative features (CFs) and parameter features (PFs), are the model inputs. The tool wear vectors ${\hat{y}}_{T_{c}-L:T_{c}}$ and ${\hat{y}}_{T_{c}+1:T_{c}^{{\mathrm{VB}}_{t}}}$ are the model outputs.

In the following, a model for the remaining tool life prediction can be trained using the dataset generated in the previous section. Figure 5 shows the model implementation according to the methodology described in Section 3.2 and Section 3.3.

The regression component is based on the Auto-sklearn library [41], enabling the implementation of the feature importance ranking algorithm (Algorithm 1). Using Auto-sklearn, CASH optimization can be performed based on ML pipelines with dedicated feature selection, restriction to importance-based feature selection methods and ensembling multiple ML pipelines. Auto-sklearn is selected due to its large model architecture and hyperparameter search space and especially its high number of included feature selection methods. A comparison between Auto-sklearn and two other state-of-the-art AutoML frameworks (LightAutoML [42] and FLAML [43])is described in Appendix C. The forecasting component is implemented using the Darts library [44]. An LSTM neural network is selected as the underlying model since LSTMs enable future features to be taken into account in time-series forecasting. Therefore, the remaining tool life prediction can be extended with prior knowledge about future machining operations as described in Section 3.2. To ensure a holistic AutoML approach, an additional wrapper is implemented around the forecaster model using the Tune library [45]. The wrapper combines the asynchronous successive halving algorithm (ASHA) [46] as a search algorithm with the tree-structured Parzen estimator [47] as a scheduler to enable the joint neural architecture and hyperparameter search.

In practical implementation, LSTMs base their predictions on the features in a limited window of length *L* from the past and do not include the entire history, as this quickly leads to intractability in the calculations. Therefore, the feature tensor $X_{T_{c}-L:T_{c}}^{\left(p\right)}$ between $T_{c}-L$ and $T_{c}$ is the input of the prediction model, as shown in Figure 5. Furthermore, the forecast must extend to the time point $T_{{\mathrm{VB}}_{t}}$ where the wear mark threshold ${\mathrm{VB}}_{t}$ is reached to enable the estimation of the remaining tool life. However, forecasting models have a fixed forecasting horizon *H*. Thus, the LSTM must be autoregressive to allow for an estimate of the remaining tool life at any time. The LSTM receives as input the tensor $X_{T_{c}-L:T_{c}^{{\mathrm{VB}}_{t}}}^{\left(f\right)}$ between $T_{c}-L$ and $T_{c}^{{\mathrm{VB}}_{t}}$ as well as the predictions of the regressor component ${\hat{y}}_{T_{c}-L:T_{c}}$ between the $T_{c}-L$ and $T_{c}$ and predicts the future tool wear ${\hat{y}}_{T_{c}+1:T_{c}^{{\mathrm{VB}}_{t}}}$ between the $T_{c}+1$ and $T_{{\mathrm{VB}}_{t}}$.

## 5. Results and Discussion

### 5.1. Dataset and Evaluation Approach

**Figure 6:**
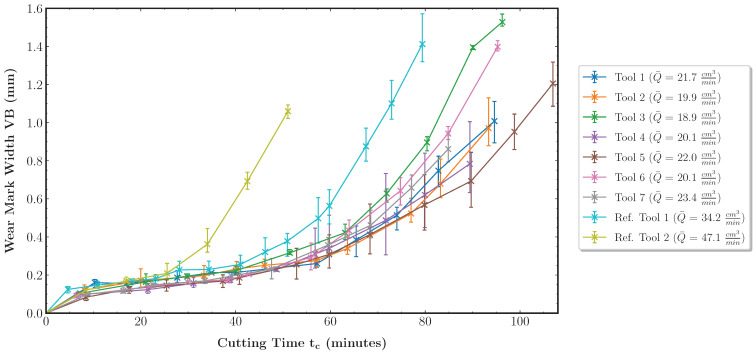
Tool wear mark width measurements and average material removal rates for the nine tools of the dataset. A cross marks the average maximum wear mark width over all cutting inserts i∈{1,2,3}. The vertical bars denote the maximum and minimum individual wear mark width values ${\mathrm{VB}}_{i}$ among the inserts.

Following the methodology introduced in Section 4.2, the dataset is generated as a foundation for the evaluations performed in the following. In total, the data of nine tools are acquired over their lifetime. Figure 6 shows the measured wear curves of the tools. In addition, the average material removal rate $\bar{Q}$ according to Equation (5) is shown per tool. A discrete number $N_{T_{c}}$ of cutting time steps $T_{c}$ represents the tool life. The total removed material volume per time step is denoted as $V_{T_{c}}$.

(5) $$\bar{Q}=\frac{1}{N_{T_{c}}}\sum_{T_{c}}\frac{V_{T_{c}}}{T_{c}}$$

Tools 1 to 7 manufacture variable pocket geometries with variable cutting parameters. This results in material removal rates between 18.9 $\frac{{\mathrm{cm}}^{3}}{\min}$ and 23.4 $\frac{{\mathrm{cm}}^{3}}{\min}$. Furthermore, the data during the lifetimes of two reference tools are acquired. Reference tool 1 manufactures variable pocket geometries under fixed cutting parameters. The cutting parameters are set to the maximum values of the intervals specified for tools 1 to 7. The maximized cutting parameters lead to an increase in $\bar{Q}$ to 34.2 $\frac{{\mathrm{cm}}^{3}}{\min}$ since the machining time of the pockets decreases. Thus, higher productivity is achieved. Reference tool 2 is applied in pure face milling based on the zig-zag strategy with fixed, maximum cutting parameters. Since only face milling is performed, the workpiece geometry can also be considered fixed. In this case, the maximum material removal rate of 47.1 $\frac{{\mathrm{cm}}^{3}}{\min}$ is achieved as no pocket milling is performed. The data from the reference tools are used to evaluate the generalization performance of the prediction models. For reference tools 1 and 2, the wear progress increases due to the increased productivity, while this is not the case for tools 1 to 7. An explanation for this is the influence of the varying workpiece geometries and, thus, loads on the tool cutting edges. Not only do the cutting parameters and the resulting machining speed affect the wear progress, but the combination with the workpiece geometry must always be considered.

**Figure 7:**
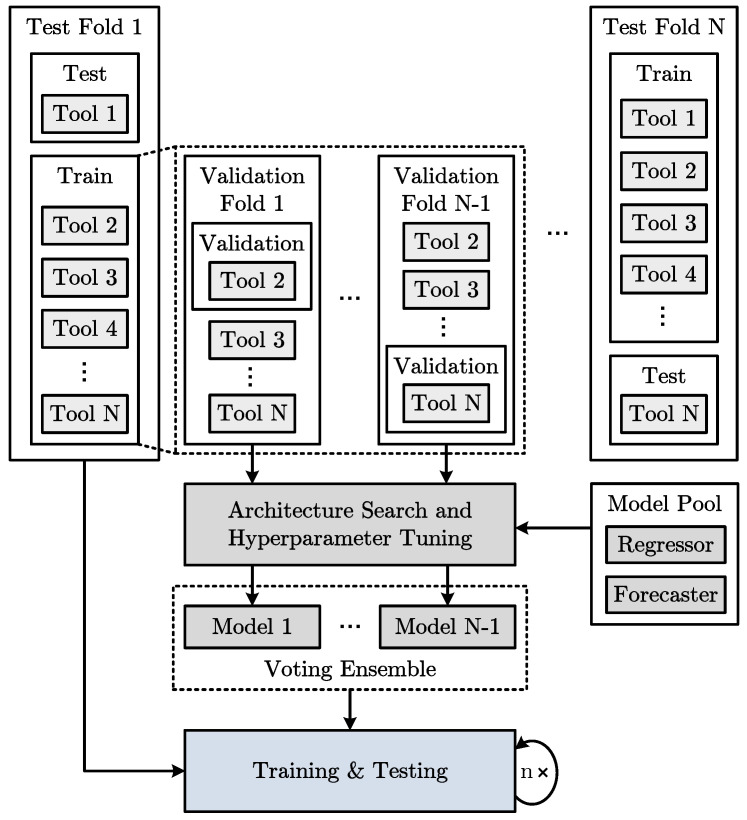
Leave-one-tool-out cross-validation and testing (LOTO-CVT) strategy for the remaining tool life prediction methodology based on a dataset with a limited number of tools *N*.

To achieve an optimal test coverage of the prediction models with a limited number of available tools and ensure their robustness, the validation and test strategy shown in Figure 7 based on the leave-one-group-out methodology is used. The strategy is denoted as leave-one-tool-out cross-validation and testing (LOTO-CVT).

The data from *N* tools are divided into training and test sets to generate the regression and forecasting models. The data of a particular test tool are excluded from model training. Each tool is used once for testing to ensure that the prediction methodology is functional for arbitrary permutations and that its performance is not just based on the random selection of individual test tools. The model architecture and hyperparameter search are then performed based on the training set containing the data of N−1 tools. Model architecture and hyperparameter configurations are sampled from a model pool. When searching for the best configuration, a search criterion is required, enabling the evaluation of the configurations and their optimization. As with training, the prediction error can be used for this purpose. However, an additional validation tool has to be kept out of the training set. The evaluation of a model using the data of the validation tool guides the search.

It is problematic that selecting a single random validation tool can overfit the models, thus misleading the architecture and hyperparameter search. Hence, each tool is used once for validation to generate a model robust to the test tool data. The resulting models of the N−1 validation folds are combined into a voting ensemble. The outputs of the models are averaged to compensate for overfitted models. After the model architecture and hyperparameter search is complete, the voting ensemble models are trained using the data from all training tools. Subsequently, the evaluation is performed based on the data of the test tool. The stochastic nature of the parameter initialization and optimization of machine learning models may lead to different model outputs for multiple training runs. Training and testing are repeated *n* times to enable reliable model quality assessment.

### 5.2. Prediction Model Evaluation

The ability of the model approach introduced in Section 3.2 to predict tool wear and remaining tool life under variable process conditions is investigated using tools 1 to 7 in the following. First, the regression component and then the overall model extended by the forecasting component are investigated. The regression component quantifies the tool condition based on a tool wear prediction up to the current time point $T_{c}$. Previous approaches rely primarily on instantaneous features derived from sensor data as an input to tool wear prediction models. The reason for this is the fixed process conditions during a tool life cycle assumed in previous work, resulting in comparable cutting processes and a direct correlation with gradual tool wear. A common approach based on vibration data, as in [30] or [34], is to perform spectral analysis of the cutting operations, with frequency bins of power or amplitude spectra representing the features. Our methodology also incorporates spectral analysis in the form of the power spectral density. However, it goes beyond that by using the cumulative features, workpiece and cutting parameters as model inputs.

We evaluate the explainable state-of-the-art approach given in [30] based on our dataset described in Section 5.1 and the LOTO-CVT strategy. In [30], the wear prediction is a classification problem based on a random forest model, which receives the frequency spectra from structure-borne sound signals acquired during cutting operations as input. The approach is transferable to our regression component since random forest models can also be used for regression problems. First, only the power spectra of the accelerometer signals are used as input to the regression model. Before the evaluation is performed using tools 1 to 7, the correlation of the power spectra with tool wear is ensured under fixed process conditions using the data from reference tool 2. We then compare the results based on the methodology described in [30] with the wear predictions of our AutoML-based regression model using the extended feature set proposed in this paper.

**Figure 8:**
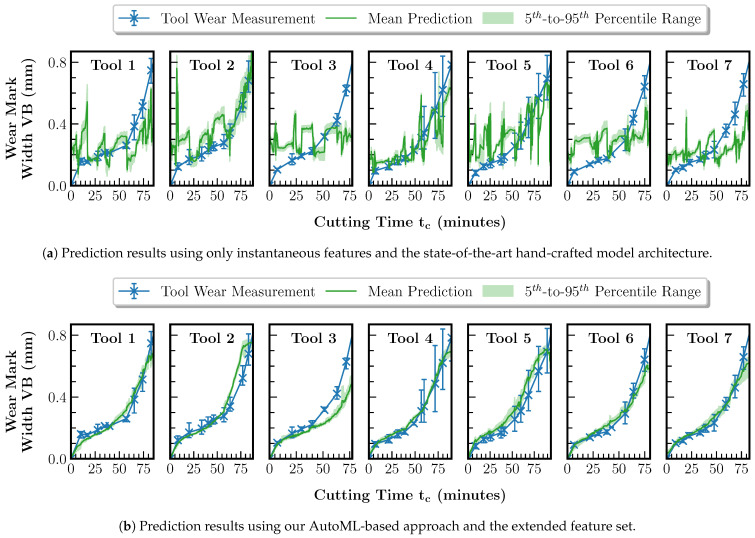
Comparison of tool wear regression results using only the instantaneous features in combination with a state-of-the-art hand-crafted model architecture and the AutoML-based tool wear regression using the extended feature set proposed in this paper. The tool’s data whose prediction results are displayed have been excluded from the training set.

For the regression model training, the Auto-sklearn environment is configured. Both the meta-learning and ensembling capabilities of Auto-sklearn are enabled. The maximum time budgets are set to 10 min for the entire CASH optimization and 30 s for training a single pipeline configuration with a memory limit of 20 GB per pipeline. The R2 score function is used as a metric for training. The training and testing steps are repeated five times according to the LOTO-CVT strategy. Since the regression is only required up to the end-of-life criterion ${\mathrm{VB}}_{t}$, the range for prediction and evaluation is limited to 0.8 mm. Figure 8 shows the comparison of the regression results. For a comprehensive error analysis, the prediction errors in terms of root-mean-square error (RMSE) and mean absolute error (MAE) over the dataset are summarized in Table 2.

Figure 8 and Table 2 show that the state-of-the-art method for tool wear prediction described in [30], which is purely based on the instantaneous spectral features, is not easily transferable to the case of variable process conditions during the tool life cycle. Estimating the wear measurement curve is only partially possible to a limited extent, as seen in Figure 8a, e.g., for tools 2 and 4. In comparison, the predictions based on our method with the extended feature set achieve a reduction in the RMSE of between 43.4 and 80.2% and in the MAE of between 54.8 and 78.8%. As seen in Figure 8b, the prediction is possible for all tools and is mainly within the measured wear value intervals of the tool cutting edges. For tool 3 only, the prediction lies outside the wear value interval starting from a cutting time of 40 min. An explanation for this could be that tool 3 has the highest material removal rate of tools 1 to 7. Thus, the wear curve represents an extreme case of the dataset and the regression model has to perform an extrapolation during inference, which is much more error-prone than an interpolation. Overall, the better performance of our method compared to purely spectral feature-based prediction can be explained by the additional features. Under variable process conditions, their influence on the signals dominates, reducing the correlation between the instantaneous features and the tool wear. Particularly, the new cumulative features allow our method to restore the comparability of the cutting operations. The feature importance is investigated in Section 5.3 to confirm this hypothesis using Algorithm 1.

**Figure 9:**
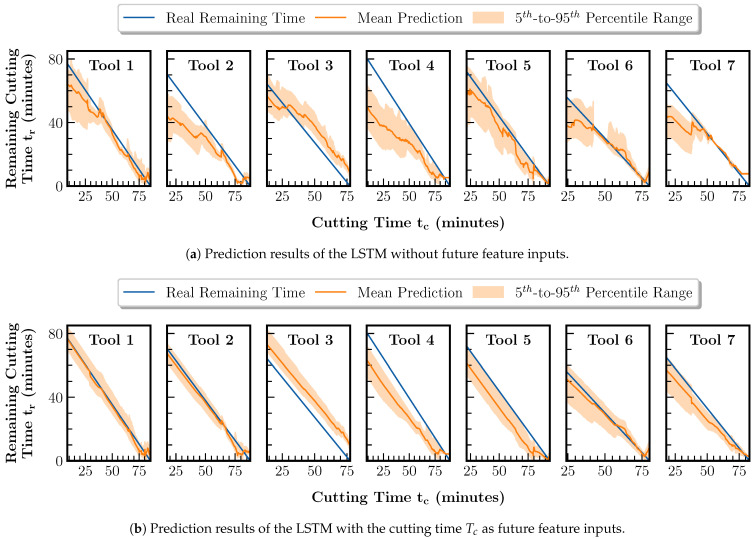
Comparison of the remaining tool life prediction results using the LSTM without future feature inputs and the LSTM with the cutting time $T_{c}$ as future feature inputs. The tool’s data whose prediction results are displayed have been excluded from the training set.

In advance, the evaluation of the remaining tool life prediction based on the previously trained regression component is performed. The goal is to analyze how the extension of the remaining tool life prediction model compared to the state-of-the-art method through the possibility of entering future feature estimates affects the predictions. For this purpose, the LSTM-based forecasting component, according to Section 4.3, is trained and tested using the data from tools 1 to 7. Based on the LSTM output, the remaining tool life is calculated using Equation (1). The forecasting component is first tuned and trained based on the LOTO-CVT strategy. The LSTM model has a single layer and a hidden dimension of 25. The length of the model input sequences between 12 and 60 samples and the output sequences between 1 and 36 samples is subject to the model tuning. Furthermore, the hyperparameters of the batch size in the range of [4,32] and learning rate in the range of $\left[10^{-5},10^{-2}\right]$ are tuned, guided by the MAE. An LSTM instance can train for a maximum of 30 epochs while early stopping is employed. In total, the training and testing of the models are repeated five times. In the testing phase, the outputs of the regression component shown in Figure 8b are input to the LSTM. Additionally, an exploration of non-spectral feature combinations as future feature inputs is performed. Figure 9 and Table 3 show the remaining tool life prediction results for the LSTM without future features and the best-performing LSTM with future features.

The remaining tool life prediction without future features has an average RMSE of 9.5 min and an MAE of 7.8 min. With future features, the RMSE is reduced by 32% to 6.5 min and the MAE by 22% to 6.1 min. The results are achieved using the total cutting time $T_{c}$ as a single future feature input. In Figure 9, the difference in prediction accuracy between the two model instances becomes evident. Without future features, the prediction is primarily inaccurate in the early stages of tool life, as shown in Figure 9a. Including the future features allows for a mostly accurate estimation of the remaining tool life at arbitrary time points. Only the predictions for tools 3, 4 and 5 in Figure 9b are characterized by a constant offset error. However, for tools 4 and 5, the predictions converge toward the real remaining time in the last 10 min of their respective lifetime. For tool 3 only, the offset remains constant until the end of its life. In this case, the offset is because the wear regression lies outside the measured wear values, as already described in the context of Figure 8b. Therefore, the regression error is propagated to the forecast and prevents the correct estimation of the future course of the wear curve. For tool 2, the maximum optimization of the prediction is achieved by an error reduction of 79% in RMSE and 78% in MAE. In addition, the dispersion of the predicted values over the entire tool life represented by the 5th-to-95th percentile range can be reduced using future features. The decreased dispersion indicates a reduction in the model uncertainty regarding the future. Overall, it can be confirmed that the remaining tool life prediction is possible under variable process conditions. In addition, an increase in accuracy and higher robustness of the prediction can be achieved by including process-describing information about future machining operations.

### 5.3. Feature Importance Analysis

**Figure 10:**
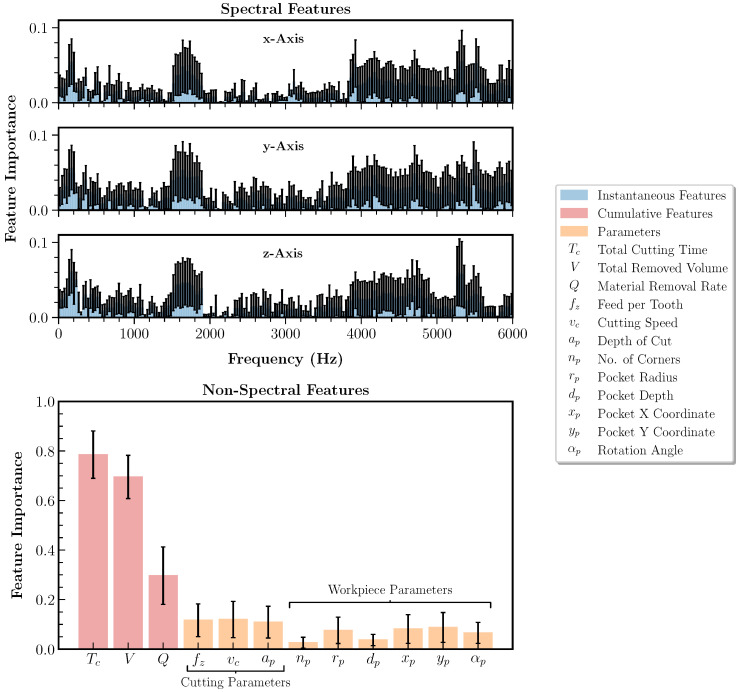
Feature importance analysis of the spectral instantaneous, cumulative and parameter features derived from the AutoML-based feature importance ranking algorithm introduced in this paper. The mean feature importance scores and their standard deviation over all tools are shown.

Based on the evaluation of the remaining tool life prediction method in the previous section, the feature importance analysis is performed in the following. The aim is to demonstrate and evaluate the feature importance ranking method according to Algorithm 1. Furthermore, it should be investigated why state-of-the-art prediction methods for fixed process conditions based on instantaneous features, such as [30], are not directly applicable to variable process conditions. Therefore, Figure 10 shows the feature importance scores derived according to Algorithm 1 for all input features of the regression model whose predictions are depicted in Figure 8b. The feature importance scores are averaged over the complete dataset, i.e., over all tools, and split by spectral and non-spectral features. The mean feature importance scores and the standard deviations are displayed.

The maximum feature importance scores of the cumulative features with mean values of 0.8, 0.7 and 0.3 for the total cutting time $T_{c}$, the total removed volume *V* and the material removal rate *Q*, respectively, indicate that they contribute more to the model decisions than the parameters or instantaneous features. The low weighting of the instantaneous spectral features supports the hypothesis that the correlation between the values of the spectra per frequency bin and the target, i.e., the tool wear, decreases due to the influence of the variable process conditions on the sensor signals. Due to their higher level of abstraction and inherent memory capability, cumulative features can maintain correlation with the target despite variable process conditions. The memory capability also distinguishes them significantly from the parameter features, which have low feature importance scores, similar to those of the instantaneous features. A detailed influence analysis of the separate feature subsets and individual high-importance features on the tool wear prediction performance can be found in Appendix D.

It has to be noted that the methods for tool wear prediction studied in this paper, i.e., the state-of-the-art method from [30] and our AutoML-based approach, rely purely on classical ML models. However, the influences of variable process parameters may be filtered out from the spectra using deep learning models, such as CNN-LSTMs, which are particularly good at representing spatio-temporal relationships. Thus, the correlation with the target could also be recovered for variable process parameters. Furthermore, the dataset used in this paper represents gradual tool wear. Abrupt tool wear, e.g., the breakage of the cutting edges due to excessive cutting forces caused by critical engagement conditions, is not included. Although the manufacturing of pocket geometries causes a variation in the engagement conditions, their influence on the sensor signals is only moderate. The influence increases for critical engagement conditions. Thus, the instantaneous features gain importance again for detecting abrupt tool wear. Moreover, the instantaneous features provide a simple wear indicator at fixed process conditions. Overall, despite their low feature importance for the dataset used in this paper, the instantaneous features are a necessary component of tool wear and tool life prediction models.

### 5.4. Generalization Performance

In the final evaluation step, the remaining tool life prediction method proposed in this paper is investigated in terms of its generalization performance. The aim is to evaluate whether a prediction of tool wear and remaining tool life is possible with increased productivity of the machining process by reducing the machining time without explicit training. Productivity is determined by the material removal rate derived from the cutting parameters feed per tooth $f_{z}$ and cutting speed $v_{c}$ defining the feed rate and the axial depth of cut $a_{p}$. Increasing the cutting parameters introduces uncertainty regarding the changing tool wear development and its impact on the workpiece quality. If the approach proposed in this paper allows transferability to increased yet unknown cutting parameter configurations, process reliability can be ensured nonetheless.

The evaluation is performed using two reference tools, reference tools 1 and 2. The data from reference tool 1 represents the test set. It is based on pocket manufacturing at fixed, maximum cutting parameters, resulting in an increase in the material removal rate and thus productivity of between 32 and 45% compared to tools 1 to 7. Two training set scenarios are distinguished to study the transferability to the variable pocket manufacturing with increased productivity:

1. **Tools 1–7 and reference tool 2**: Knowledge of the target wear curve for variable pocket manufacturing using variable cutting parameters and of the wear curve for face milling using fixed, maximum cutting parameters.
2. **Tools 1–7**: Knowledge of the target wear curve for variable pocket manufacturing using variable cutting parameters only.

**Figure 11:**
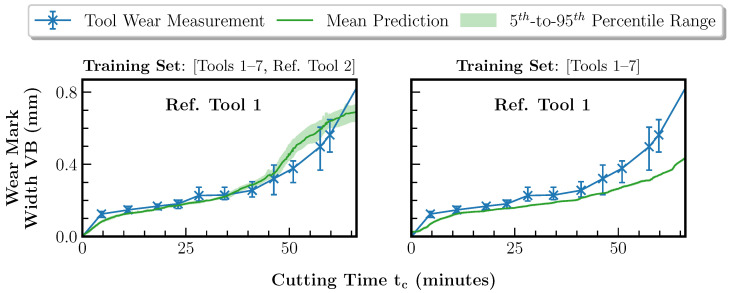
Comparison of the tool wear regression results using the two training sets and the data of reference tool 1 as the test set. The data of reference tool 1 have been excluded from the training set.

The regression and forecasting components are trained with both training sets. Figure 11 and Table 4 show the tool wear prediction results of the regression component.

Based on training set 1, the prediction results lie mostly within the wear measurement intervals, leading to an RMSE of 0.054 mm and an MAE of 0.041 mm. In the case of training set 2, excluding reference tool 2, the same behavior as for tool 3 in Figure 8b is obtained. Reference tool 1 represents the tool life cycle with the maximum material removal rate and the fastest wear progress. This leads to a significant underestimation of the wear curve with an RMSE of 0.108 mm and an MAE of 0.078 mm. The result supports the hypothesis that the regression component of our approach is not able to extrapolate the wear curve. With additional knowledge of the wear curve for face milling (training set 1) with a material removal rate of 47.1 $\frac{{\mathrm{cm}}^{3}}{\min}$ exhibiting faster wear progress than reference tool 1, the regression model performs an interpolation, leading to a feasible prediction. Furthermore, the investigation can also verify the high feature importance scores of the three cumulative features $T_{c}$, *V* and *Q* noted in Section 5.3. The material removal rate and thus the two parameters $T_{c}$ and *V* define the wear progress in the considered scenario of gradual tool wear and are thus crucial for the regression. Overall, the transferability of the regression component to pocket milling at increased productivity is given, provided that the task represents an interpolation.

**Figure 12:**
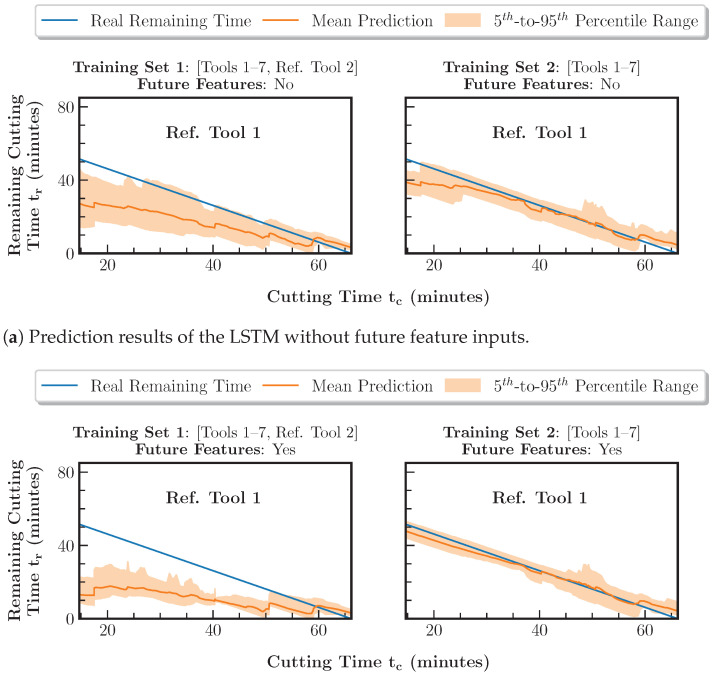
Comparison of the remaining tool life prediction results using the two training sets combined with the LSTM without future feature inputs and the LSTM with the cutting time $T_{c}$ as future feature inputs. The data of reference tool 1 have been excluded from the training set.

For evaluating the forecasting component, the output of the regression model based on training set 1 is used as the LSTM input in the testing phase. Furthermore, an exploration of non-spectral feature combinations as future feature inputs is performed, as in Section 5.2. The results of the remaining tool life prediction for reference tool 2 are shown in Figure 12 and Table 5.

Figure 12 shows that, based on training set 2, the prediction is feasible with an RMSE of 4.9 min and an MAE of 3.5 min. In contrast to the regression component, which provides a feasible prediction based on training set 1, the forecasting component trained with training set 1 significantly underestimates the remaining tool life with an RMSE of 14.9 min and an MAE of 12.7 min. An explanation for this behavior is the sensitivity of the LSTM to the characteristic temporal wear curve progression during pocket manufacturing. In this context, face milling represents a modified workpiece geometry and, as part of the training set, mitigates the transferability of the LSTM to pocket manufacturing with different cutting parameter configurations. When the LSTM input is extended to include the future features, as shown in Figure 12b, the prediction for the model based on training set 1 deteriorates with an RMSE of 22.8 min and an MAE of 19.3 min. For the feasible LSTM based on training set 2, the behavior already observed in Figure 9b for tools 1 to 7 repeats. In the early stage of the tool life up to a cutting time of 40 min, the prediction accuracy can be increased and the uncertainty can be reduced. This results in minimum values of the RMSE of 2.2 min and the MAE of 1.8 min.

Overall, the remaining tool life prediction approach introduced in this paper allows transferability to pocket manufacturing at increased, previously unknown parameter configurations, thus ensuring process reliability at increased productivity. The evaluation in this paper refers to a specific combination of workpiece material and tool type within the milling process. Due to its general architecture, the remaining tool life prediction methodology applies to other combinations and machining processes without a loss of generality. However, the extent to which the model generated in this paper needs to be re-trained depends on the distance of the resulting data distributions.

## 6. Conclusions and Future Work

In this paper, a new method for predicting remaining tool life under the variable process conditions of individualized machining production was presented. The method is criterion-based, i.e., it inherently uses a dedicated variable to represent the tool condition based on which the remaining tool life is determined. In contrast to the traditional approach, where the remaining tool life models are created manually, the introduced method is based on AutoML. The model decisions depending on feature importance scores are then extracted and visualized. Domain experts from the field of machining are thus enabled to develop, validate and optimize remaining tool life models without extensive ML knowledge. The AutoML-based modeling procedure is complemented by a feature set optimized for prediction in individualized production scenarios, obtained purely using non-invasive vibration-based process monitoring. A prototypical vibration sensor system was implemented using an FPGA-SoC-based hardware platform on a five-axis indexed milling center for evaluation. The setup was used to generate a dataset representing gradual tool wear under continuous variation in workpiece geometries and cutting parameters. Based on the dataset, several experiments were conducted to evaluate the method for predicting the remaining tool life.

The experiments reveal that a transfer of manually created remaining tool life prediction models for the case of series production, i.e., constant process conditions, fails due to the input features used in previous work. In order to enable a feasible prediction of the remaining tool life for individualized production, methods extracting features invariant to variable process conditions and preserving the correlation to the tool condition are necessary. Our methodology improves the prediction accuracy over manually created state-of-the-art models by up to 80% with an average MAE of 6.1 min. This corresponds to an accuracy of 7% of the average tool life throughout the dataset. Furthermore, it can be shown that integrating prior knowledge about future machining operations improves the accuracy of the remaining tool life prediction by up to 22% and increases its robustness. The consideration of prior knowledge in the models can also be exploited to perform process optimization, e.g., targeting productivity through virtual exploration of the effect of yet-unknown cutting parameters on the tool condition without affecting process reliability. Thus, the remaining tool life prediction enables the full utilization of the tool life and increased plannability at the job-shop level in individualized production. Overall, the remaining tool life prediction leverages Pareto optimization in manufacturing, targeting product quality, tool costs and productivity.

The AutoML-based modeling approach presented in this paper is, at its core, universally applicable to data- and criterion-based remaining life predictions of technical systems. Nevertheless, the overall methodology is highly specialized due to the domain-specific feature extraction. In order to achieve transferability to other applications, the methodology will be extended in the future using a general feature extraction mechanism, e.g., using CNNs. Furthermore, the dataset generated in the paper represents only gradual tool wear. However, a fully comprehensive tool life model must also handle abrupt tool wear, e.g., due to critical tool engagement conditions. An extension of the dataset will be necessary in the future to investigate abrupt tool wear.

## Figures and Tables

**Table 1:** Components of the feature set used as input to the prediction methodology, divided into instantaneous, cumulative and parameter features.

Feature Subset	Parameter	Description
Instantaneous	$P_{\mathrm{xx}}^{\left(0–6\;\mathrm{kHz}\right)}$	Tensor, including the bins of the power spectra between 0 and 6 kHz for the three accelerometer channels (x, y, z)
Cumulative	$T_{c}$	Total cutting time
	*V*	Total removed material volume
	*Q*	Material removal rate
Cutting Parameters	$f_{z}$	Feed per tooth
	$v_{c}$	Cutting speed
	$a_{p}$	Axial depth of cut
Workpiece Parameters	$c_{p}$	No. of pocket corners
	$r_{p}$	Pocket radius
	$d_{p}$	Pocket depth
	$\left(x_{p},y_{p}\right)$	Pocket center point
	$\alpha_{p}$	Rotation angle

**Table 2:** Comparison of tool wear regression errors using only the instantaneous features in combination with a state-of-the-art hand-crafted model architecture and the AutoML-based tool wear regression using the extended feature set proposed in this paper.

Method	Metric	Prediction Errors per Test Tool (mm)							
		Tool 1	Tool 2	Tool 3	Tool 4	Tool 5	Tool 6	Tool 7	Mean
Hand-crafted	RMSE	0.169	0.140	0.189	0.131	0.199	0.182	0.162	0.167
	MAE	0.133	0.102	0.155	0.097	0.147	0.143	0.132	0.130
Ours	RMSE	**0.038**	**0.068**	**0.107**	**0.026**	**0.066**	**0.065**	**0.041**	**0.059**
	MAE	**0.031**	**0.046**	**0.070**	**0.021**	**0.055**	**0.045**	**0.028**	**0.042**

**Table 3:** Comparison of the remaining tool life prediction errors using the LSTM with no future feature inputs and the LSTM with the cutting time $T_{c}$ as future feature inputs.

Future Features	Metric	Prediction Errors per Test Tool (Minutes)							
		Tool 1	Tool 2	Tool 3	Tool 4	Tool 5	Tool 6	Tool 7	Mean
No	RMSE	5.7	12.9	**7.9**	16.5	**6.9**	7.1	9.6	9.5
	MAE	4.7	10.8	**7.2**	14.0	**6.0**	4.9	6.5	7.8
Yes	RMSE	**2.0**	**2.7**	9.9	**12.2**	9.8	**3.0**	**6.1**	**6.5**
	MAE	**1.6**	**2.4**	9.9	**11.4**	9.4	**2.6**	**5.6**	**6.1**

**Table 4:** Comparison of the tool wear regression errors using the two training sets and the data of reference tool 1 as the test set.

Metric	Prediction Errors for Ref. Tool 1 per Training Set (mm)	
	Training Set 1: Tools 1–7 and Ref. Tool 2	Training Set 2: Tools 1–7
RMSE	**0.054**	0.108
MAE	**0.041**	0.078

**Table 5:** Comparison of the remaining tool life prediction errors using the two training sets combined with the LSTM with no future feature inputs and the LSTM with the cutting time $T_{c}$ as future feature inputs.

Future Features	Metric	Prediction Errors for Ref. Tool 1 per Training Set (Minutes)	
		Training Set 1: Tools 1–7 and Ref. Tool 2	Training Set 2: Tools 1–7
No	RMSE	14.9	4.9
	MAE	12.7	3.5
Yes	RMSE	22.8	**2.2**
	MAE	19.3	**1.8**

**Figure A1:**
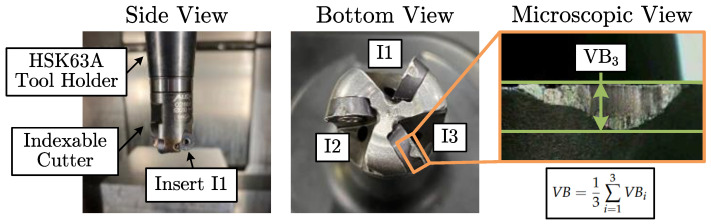
Measurement procedure of the individual wear mark width ${\mathrm{VB}}_{i}$ per cutting insert *i*.

**Figure A2:**
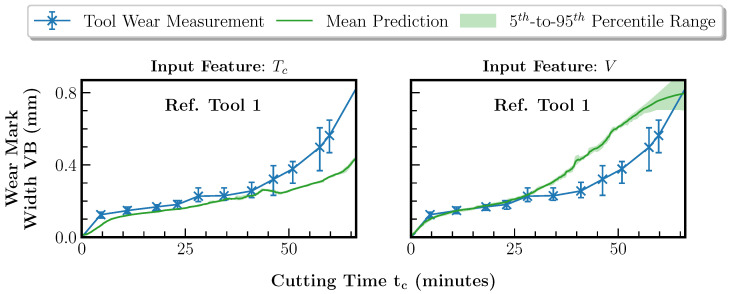
Comparison of the tool wear regression results using the individual high-importance features $T_{c}$ and *V* based on the training set composed of tools 1 to 7 and reference tool 2. The data of reference tool 1 are the test set and are thus excluded from the training set.

## Data Availability

The data presented in this study are available on request from the corresponding author. The data are not publicly available due to privacy reasons.

## Appendix A. Wear Mark Width Measurement

The target parameter of the dataset introduced in this paper is the wear mark width VB. The VB is acquired using a video measuring microscope according to the procedure shown in Figure A1.

First, the maximum wear mark width ${\mathrm{VB}}_{i}$ per insert $i\in \{1,\ldots ,z_{c}\}$ is measured. $z_{c}$ is the number of inserts of the cutting tool. Then, the average over the maximum wear mark widths is calculated according to Equation (A1) to obtain the target value VB.

(A1) $$\mathrm{VB}=\frac{1}{z_{c}}\sum_{i=1}^{z_{c}}{\mathrm{VB}}_{i}$$

## Appendix B. Milling Process Details

Table A1 summarizes the specifications of the reference milling process developed in this paper for the dataset generation. In addition to the specifications, Figure A1 shows the used tool holder, cutter and cutting inserts. A tool instance of the dataset refers to a set of three cutting inserts.

**Table A1:** Fixed and variable parameters of the reference milling process for the dataset generation.

Category	Parameter	Unit	Range/Value
Cutting Process	Feed per Tooth ($f_{z}$)	mm	[0.4,0.7]
	Cutting Speed ($v_{c}$)	$\frac{\mathrm{mm}}{s}$	[170,200]
	Axial Cut Depth ($a_{p}$)	mm	[0.5,0.8]
	Radial Cut Depth ($a_{e}$)	mm	*R*
Pocket Geometry	Corners ($c_{p}$)	-	[3,9]
	Radius ($r_{p}$)	mm	[30,100]
	Depth ($d_{p}$)	mm	[1,10]
	Position $\left(x_{p},y_{p}\right)$	mm	[50,150], [50,150]
	Rotation Angle ($\alpha_{p}$)	${}^{\circ }$	[0,360]
Milling Tool	Type	-	Indexable
	Tool Shape	-	Toroidal
	Edge Shape	-	Circular
	Number of Teeth ($z_{c}$)	-	3
	Cutter Radius (*R*)	mm	10
	Edge Radius (*r*)	mm	4
	End-of-life Threshold (${\mathrm{VB}}_{t}$)	mm	0.8
Workpiece	Dimensions (l,w,h)	mm	200, 200, 200
	Material	-	X155CrVMo12-1 (DIN 1.2379)
	Consecutive Pockets ($N_{p}$)	-	4

## Appendix C. AutoML Framework Analysis

Before evaluating the remaining tool life prediction approach introduced in this paper, an AutoML framework must be selected as a core component of the methodology. For this purpose, a comparison between the three state-of-the-art AutoML systems, Auto-sklearn [41], LightAutoML [42] and FLAML [43], is performed. Table A2 compares the size of the search space of the systems based on the included feature selection methods and ML model types for regression tasks. LightAutoML has the smallest search space, directly followed by FLAML. Auto-sklearn offers the largest search space of the AutoML systems, with 13 feature selection methods and 12 model types. Thus, the space of not only the model architectures but also the tunable hyperparameters is larger compared to the other frameworks.

**Table A2:** Comparison of the search space size with respect to the number of feature selection methods and ML model types between the state-of-the-art frameworks Auto-sklearn [41], LightAutoML [42] and FLAML [43].

AutoML Framework	Search Space Size (Number of Included Methods)	
	Feature Selection	Model Types
LightAutoML	2	2
FLAML	3	6
Auto-sklearn	13	12

The frameworks are compared quantitatively based on the dataset generated in this paper in the context of tool wear regression. The entire feature set, as described in Section 5.2, is used as model input for the tool wear regression, and the model training is performed with a maximum time budget of 10 min. The prediction errors and search runtimes are shown in Table A3 and Table A4, respectively.

**Table A3:** Comparison of tool wear regression errors using the full input feature set and the state-of-the-art frameworks Auto-sklearn [41], LightAutoML [42] and FLAML [43]. Every run is performed with a maximum search time budget of 10 min.

Method	Metric	Prediction Errors per Test Tool (mm)							
		Tool 1	Tool 2	Tool 3	Tool 4	Tool 5	Tool 6	Tool 7	Mean
Light AutoML	RMSE	**0.033**	0.050	**0.079**	**0.022**	**0.051**	**0.064**	0.036	**0.048**
	MAE	**0.027**	0.035	**0.054**	**0.016**	**0.042**	0.043	0.026	**0.035**
FLAML	RMSE	0.035	**0.044**	0.086	0.023	0.062	0.065	**0.034**	0.050
	MAE	0.028	**0.032**	0.057	0.017	0.047	**0.041**	**0.022**	**0.035**
Auto-sklearn	RMSE	0.038	0.068	0.107	0.026	0.066	0.065	0.041	0.059
	MAE	0.031	0.046	0.070	0.021	0.055	0.045	0.028	0.042

**Table A4:** Comparison of the final search runtimes required by the state-of-the-art frameworks Auto-sklearn [41], LightAutoML [42] and FLAML [43].

Method	Search Time (s)							
	Tool 1	Tool 2	Tool 3	Tool 4	Tool 5	Tool 6	Tool 7	Mean
Light AutoML	185.0	**185.1**	**186.6**	**186.2**	**185.7**	**186.3**	186.5	**185.9**
FLAML	**165.2**	308.2	280.8	214.6	238.6	214.8	**177.8**	228.6
Auto-sklearn	600.0	600.00	600.0	600.0	600.0	600.0	600.0	600.0

Table A3 shows that LightAutoML achieves the best regression result over the tools of the dataset with an RMSE of 0.048 mm and an MAE of 0.035 mm. At the same time, the mean search runtime is minimal at 3.1 min, according to Table A4. FLAML follows LightAutoML with an RMSE of 0.050 mm, an MAE of 0.035 mm and a mean search runtime of 3.8 min. Auto-sklearn has the maximum amount of errors with an RMSE of 0.059 mm and an MAE of 0.042 mm. However, the regression errors of the AutoML systems vary only slightly overall. Due to the size of the search space, Auto-sklearn uses the entire time budget of 10 min for the CASH optimization. LightAutoML and FLAML apply early stopping by default.

The evaluation results coincide with the size of the respective search spaces. A smaller number of possible model architectures and tunable hyperparameters allows feasible solutions to be found faster. However, to achieve a robust and reliable explainability of the models using the feature importance-based method according to Algorithm 1, a high degree of diversity of the model pipelines and the feature selection procedures is beneficial. Since the regression errors of the AutoML systems vary only slightly and further minimization can be achieved by a larger time budget of the CASH optimization, Auto-sklearn is therefore used in this paper due to the size of the search space.

## Appendix D. Feature Subset Comparison

In addition to the feature importance analysis in Section 5.3, this section investigates the tool wear regression performance depending on the feature subsets (instantaneous, cumulative and parameters) introduced in this paper. Therefore, Auto-sklearn is configured as described in Section 5.2. Table A5 shows the prediction errors for the instantaneous, cumulative and parameter features. The prediction errors for the instantaneous features are copied from Table 2 for comparison.

**Table A5:** Comparison of tool wear regression errors using only the instantaneous, cumulative and parameter feature subsets.

Feature Subset	Metric	Prediction Errors per Test Tool (mm)							
		Tool 1	Tool 2	Tool 3	Tool 4	Tool 5	Tool 6	Tool 7	Mean
Instantaneous	RMSE	0.169	0.140	0.189	0.131	0.199	0.182	0.162	0.167
	MAE	0.133	0.102	0.155	0.097	0.147	0.143	0.132	0.130
Cumulative	RMSE	**0.036**	**0.072**	**0.061**	**0.032**	**0.054**	**0.066**	**0.025**	**0.049**
	MAE	**0.030**	**0.047**	**0.041**	**0.025**	**0.042**	**0.044**	**0.017**	**0.035**
Parameters	RMSE	0.244	0.153	0.171	0.202	0.235	0.277	0.190	0.210
	MAE	0.196	0.125	0.153	0.165	0.195	0.216	0.152	0.172

The regression model based on the cumulative features $T_{c}$, *V* and *Q* with an RMSE of 0.049 mm and an MAE of 0.035 mm performs best. The RMSE and MAE are both 17% lower than for the model based on the entire feature set, as shown in Table 2. Moreover, Auto-sklearn thus achieves approximately the same result as LightAutoML in Table A3. Overall, the particular importance of the cumulative features for the model performance, which can be seen in Figure 10, is confirmed. Based on the parameters, the regression model exhibits the worst performance with an RMSE of 0.210 mm and an MAE of 0.172 mm. This can be explained by the fact that the parameters do not correlate with the target. Therefore, their importance in Figure 10 is also consistently low. In the future, however, there is the potential to extract further wear-correlated features by combining the instantaneous features and the parameters. Since the instantaneous features significantly depend on the parameters, filtering these influences may allow the wear influence to be isolated.

As identified in Section 5.3, the total cutting time $T_{c}$, the removed material volume *V* and the material removal rate *Q* have the highest feature importance scores. Therefore, in addition to examining the feature subsets above, the prediction performance is evaluated based on the individual high-importance features. Table A6 summarizes the results.

**Table A6:** Comparison of tool wear regression errors using only individual high-importance features.

Feature	Metric	Prediction Errors per Test Tool (mm)							
		Tool 1	Tool 2	Tool 3	Tool 4	Tool 5	Tool 6	Tool 7	Mean
$T_{c}$	RMSE	**0.035**	0.046	**0.059**	0.035	**0.054**	**0.058**	**0.026**	**0.045**
	MAE	0.029	0.034	**0.041**	0.027	**0.042**	**0.037**	**0.018**	**0.033**
V	RMSE	0.042	**0.037**	0.117	**0.031**	0.080	0.077	0.057	0.063
	MAE	**0.026**	**0.029**	0.075	**0.024**	0.065	0.043	0.050	0.045
Q	RMSE	0.207	0.171	0.202	0.164	0.208	0.195	0.211	0.194
	MAE	0.185	0.129	0.137	0.134	0.181	0.152	0.180	0.157

With an RMSE of 0.045 mm and an MAE of 0.033 mm, the model trained with the feature $T_{c}$ exhibits the best performance. The errors are 8% (RMSE) and 6% (MAE) lower than for the model based on the entire cumulative feature set shown in Table A5. This result suggests that only a single feature is needed. However, considering the generalization scenario according to Section 5.4 based on the individual features, it can be seen that this does not hold. Therefore, Figure A2 shows the wear prediction based on training dataset 1, as described in Section 5.4.

The model trained using $T_{c}$ underestimates the wear curve of reference tool 1 with an RMSE of 0.110 mm and an MAE of 0.076 mm. The model trained using *V* overestimates the wear curve with an RMSE of 0.142 mm and an MAE of 0.104 mm. As shown in Section 5.4, a robust and generalizable tool wear model as the foundation for the remaining tool life prediction can only be trained based on the combination of the high-importance features.
